# The HIV-1 Rev Protein Enhances Encapsidation of Unspliced and Spliced, RRE-Containing Lentiviral Vector RNA

**DOI:** 10.1371/journal.pone.0048688

**Published:** 2012-11-01

**Authors:** Bastian Grewe, Katrin Ehrhardt, Bianca Hoffmann, Maik Blissenbach, Sabine Brandt, Klaus Überla

**Affiliations:** Department of Molecular and Medical Virology, Ruhr-University Bochum, Bochum, Germany; McGill University AIDS Centre, Canada

## Abstract

**Background:**

During the RNA encapsidation process of human immunodeficiency virus (HIV) viral genomic, unspliced RNA (gRNA) is preferentially incorporated into assembling virions. However, a certain amount of spliced viral transcripts can also be detected in viral particles. Recently, we observed that nuclear export of HIV and lentiviral vector gRNA by Rev is required for efficient encapsidation. Since singly-spliced HIV transcripts also contain the Rev-response element (RRE), we investigated if the encapsidation efficiency of RRE-containing spliced HIV-vector transcripts is also increased by the viral Rev protein.

**Findings:**

Starting with a lentiviral vector imitating the splicing pattern of HIV, we constructed vectors that express an unspliced transcript either identical in sequence to the singly-spliced or the fully-spliced RNA of the parental construct. After transfection of the different lentiviral vectors cytoplasmic and virion-associated RNA levels and vector titers were determined in the presence and absence of Rev. Rev enhanced the infectious titer of vectors containing an RRE 6 to 37-fold. Furthermore, Rev strongly increased encapsidation efficiencies of all RRE-containing transcripts up to 200-fold. However, a good correlation between encapsidation efficiency and lentiviral vector titer could only be observed for the gRNA. The infectious titer of the vector encoding the fully-spliced RNA without RRE as well as the encapsidation efficiency of all transcripts lacking the RRE was not influenced by Rev. Interestingly, the splicing process itself did not seem to interfere with packaging, since the encapsidation efficiencies of the same RNA expressed either by splicing or as an unspliced transcript did not differ significantly.

**Conclusions:**

Rev-mediated nuclear export enhances the encapsidation efficiency of RRE-containing lentiviral vector RNAs independently of whether they have been spliced or not.

## Introduction

Encapsidation of the genomic RNA (gRNA) of human immunodeficiency virus type 1 (HIV-1) is mediated by a specific interaction between the viral Gag protein and an RNA structure in the 5′ untranslated region (5′UTR) called encapsidation signal or Psi (Ψ). This association leads to incorporation of gRNA dimers into Gag/GagPol particles. Whereas the core encapsidation signal is composed of 110 nt partially overlapping the *gag* start codon it is known that sequences up- and downstream of this sequence also influence the encapsidation efficiency. All in all the entire 5′UTR (335 nt) and approximately 300 nt of *gag* are important for packaging (reviewed in [1]). Complex alternative splicing of the genomic transcript of HIV-1 generates more than 30 different RNAs that can be divided in singly-spliced and fully-spliced transcripts [2], [3]. All spliced RNAs have in common that the major splice donor (splice donor 1, SD1) is fused to a downstream splice acceptor site (SA) [2]–[4]. Since SD1 is localized in the core encapsidation signal, 46 nt preceding the *gag* start codon together with the entire *gag* sequence are removed in the course of splicing. As a consequence, the first highly structured 289 nt of the 5′UTR are present in all spliced viral RNAs. Although the gRNA is highly enriched in viral particles, a small but significant amount of spliced viral RNA species is also packaged specifically [5]. High amounts of spliced viral RNA could be detected in virus particles isolated from patients under highly active anti-retroviral therapy [6]. Under *in vitro* conditions Gag was able to bind to the 5′ end present in all viral RNAs with high affinity [7]. In cell culture based assays the polyA RNA stem loop emerged as a critical determinant for packaging of spliced RNAs [8]. Furthermore, reduction of virion-associated gRNA levels by targeted deletions in the encapsidation signal or mutation of Gag is accompanied by an increased amount of encapsidated spliced RNAs [8], [9]. Additional evidence for packaging of spliced RNAs was obtained when reverse transcribed cDNA corresponding to spliced viral RNAs was detected in HIV-1 infected cells [10], [11]. This indicates that viral particles containing spliced RNA may even be infectious.

The viral Rev protein allows nuclear export of unspliced and singly-spliced HIV transcripts via interaction with an RNA structure called Rev-response element (RRE). Recently, we discovered that a Rev-mediated export from nucleus to cytoplasm is essential for a highly efficient encapsidation process of lentiviral vector and proviral gRNA [12]–[14]. Furthermore, the presence of an RRE in murine leukemia virus gRNA was shown to increase packaging into HIV particles in the presence of Rev [15]. Since singly-spliced HIV RNAs also contain the RRE, we decided to study the influence of Rev on encapsidation of spliced HIV-1-derived vector RNA.

## Results and Discussion

### Construction of lentiviral vectors

**Figure 1:**
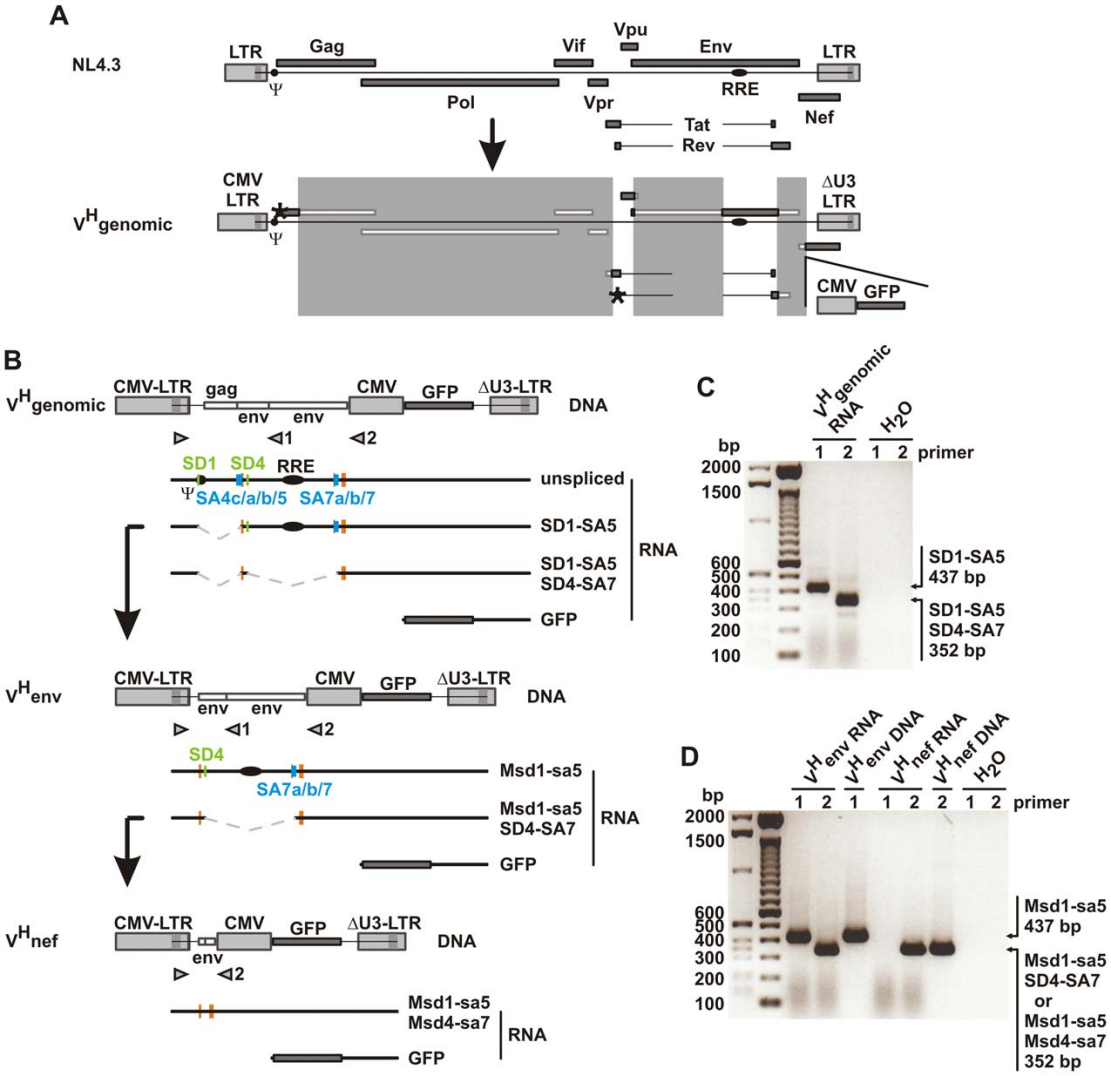
Construction of lentiviral vectors. A) The HIV-1 provirus NL4.3 and the HIV-1-vector V^H^genomic are shown. Large parts of the *gag, pol* and *env* genes are deleted in V^H^genomic (see white bars in the deleted regions marked by shaded areas). The remaining *gag* sequence contains parts of the encapsidation signal (Psi, Ψ) and the *env* fragments contain splicing regulatory elements as well as the RRE. Due to deletions (shaded squares) and frameshift mutations (black asterisks in *gag* and *rev*) no viral genes are expressed from V^H^genomic. Both vectors are drawn to scale. B) Schematic representation of the lentiviral vectors V^H^genomic, V^H^env and V^H^nef. The intron between SD1 and SA5 or the introns between SD1 and SA5 and between SD4 and SA7 were deleted from V^H^genomic in V^H^env or V^H^nef, respectively. Unspliced and spliced transcripts with splice sites (5′ splice sites in green and 3′ splice sites in blue) and *cis*-acting splicing regulatory elements (in orange) are shown. Please note that the unspliced Msd1-sa5 RNA of V^H^env is identical in sequence to the singly-spliced SD1-SA5 RNA of V^H^genomic. Furthermore, the unspliced Msd1-sa5+Msd4-sa7 RNA of V^H^nef is identical to the fully-spliced SD1-SA5+SD4-SA7 RNA of V^H^genomic and the singly-spliced Msd1-sa5+SD4-SA7 RNA of V^H^env. Arrowheads represent RT-PCR primers. C) and D) After cotransfection of lentiviral vectors with *tat* and *rev* expression plasmids into HEK293T cells cytoplasmic RNA was isolated and analyzed by RT-PCR with primer pairs depicted in figure 1B. Agarose gel electrophoretic analyses of PCR products are shown. The amplification products were sequenced to verify splicing between the indicated splice sites.

The parental lentiviral vector HIV-CS-CG [16], [17] contains the major splice donor (SD1) and the splice acceptor sites 7a, 7b and 7 surrounded by *cis*-acting splicing regulatory sequences (intron splicing silencer, exon splicing enhancer 2 and 3, exon splicing silencer 3a and 3b). In order to imitate the splicing pattern of HIV-1 we inserted a 345 bp fragment from NL4.3Re [18] between these splice sites encompassing splice acceptor sites 4a, 4b, 4c and 5, splice donor site 4 as well as the *cis*-acting regulatory sequence GAR (an exon splicing enhancer). The resulting plasmid V^H^genomic and its transcripts are depicted in figure 1A and B. Two days after cotransfection of this construct together with *rev* and *tat* expression plasmids into HEK293T cells cytoplasmic RNA was extracted and analyzed by RT-PCR. Fragments corresponding to singly-spliced and fully-spliced RNAs were detectable (figure 1C). The unspliced RNA was not detected in these experiments because short elongation times were used to specifically detect the spliced transcripts. Sequencing of the obtained fragments verified the expected fusion of SD1 with SA5 for the singly-spliced RNA and an additional splicing process between SD4 and SA7 in the fully-spliced RNA (data not shown). These also represent the predominant splicing events for the wild type virus leading to its *env1* and *nef2* transcripts [2]. The intron between SD1 and SA5 was removed from V^H^genomic to generate the vector V^H^env encoding the singly-spliced RNA of V^H^genomic as an unspliced transcript (figure 1B). The V^H^nef vector contains an additional deletion of the intron between SD4 and SA7. Thus, it encodes the fully-spliced RNA of V^H^genomic as an unspliced transcript (figure 1B). After cotransfection of V^H^env or V^H^nef in combination with *rev* and *tat* expression plasmids RT-PCR of cytoplasmic RNA detected transcripts of the expected lengths (figure 1D). Sequence analyses of the amplicons further confirmed that the expected transcripts were indeed expressed (figure 1C and D and data not shown).

### Determination of vector titers

**Figure 2:**
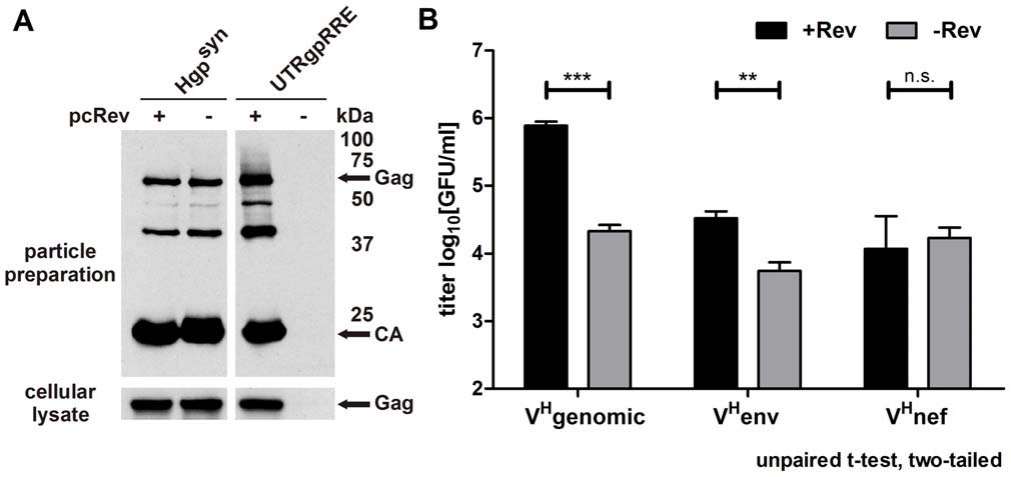
Rev-dependency of the infectious lentiviral vector titer. A) Cellular lysates and viral particles were harvested two days after transfection of HEK293T cells and were analyzed by an anti-CA Western Blot. The expression plasmid UTRgpRRE contains wild type *gag/gagpol* gene sequences combined with a part of the viral 5′UTR and the RRE. The Rev-independent *gag/gagpol* expression plasmid Hgp^syn^ encodes proteins with wild type amino acid sequences but the gene sequence is dramatically altered due to codon-optimization. B) HEK293 cells were infected with supernatants containing VSV-G pseudotyped lentiviral vectors produced in the presence or absence of Rev. Constant high Gag/GagPol protein levels were provided during vector production by cotransfection of the Rev-independent codon-optimized expression plasmid Hgp^syn^. Two days later green fluorescent cells were counted to obtain the infectious titer as GFP forming units per ml of cell culture supernatant (GFU/ml). Titer of the negative control without VSV-G and Gag/GagPol was below 50 GFU/ml (data not shown). Mean values with SEM (standard error of mean) of log10 transformed results obtained in at least 4 independent experiments are shown. Statistical analysis was performed with an unpaired two-tailed t-test with 95% confidence interval. ***, p≤0.001; **, p≤0.01; *, p≤0.05; n.s., not statistically significant.

Infectious particles were produced by transient cotransfection of HEK293T cells with the lentiviral vector V^H^genomic or V^H^env or V^H^nef together with expression plasmids for *tat*, vesicular stomatitis virus G protein (VSV-G) and *gag/gagpol* in the absence or presence of a *rev* expression plasmid. Constant and high Gag/GagPol levels were provided by the codon-optimized and therefore Rev-independent *gag/gagpol* expression plasmid Hgp^syn^. Similar amounts of Gag/GagPol are produced after cotransfection of Hgp^syn^ with or without a *rev* expression plasmid (figure 2A and [12], [13], [18]). Furthermore, Gag/GagPol levels were comparable to those detected after transfection of the corresponding subgenomic wild type *gag/gagpol* control expression plasmid UTRgpRRE in the presence of Rev. In addition, similar protein processing patterns and budding efficiencies could be demonstrated (figure 2A and [12], [13], [18]). The infectious titers of supernatants harvested two days after transfection were determined on HEK293 cells by quantifying the number of GFP positive cells two days after infection (figure 2B). The lentiviral vector V^H^genomic showed a mean titer of 7.7×10∧5 GFU/ml very similar to the parental vector V^H^ ([13] and data not shown). Omitting Rev reduced the titer 37-fold. Although transcripts expressed from V^H^env and V^H^nef lack the intron between SD1 and SA5 and therefore the 3′ part of the encapsidation signal they do contain all elements necessary for a successful RT reaction (primer binding site, 5′ and 3′ R region, central polypurine tract) and integration (wild type 5′ and 3′ ends after RT reaction). Consequently, two days after infection a mean titer of 3.3×10∧4 and 1.2×10∧4 GFU/ml in the presence of Rev could be detected for V^H^env and V^H^nef, respectively (figure 2B). The infectious titer of V^H^env was 6-fold reduced in the absence of Rev indicating that Rev is important for the production of infectious particles with V^H^env. As expected, Rev did not influence the titer of V^H^nef lacking the RRE. An alternative explanation for the *gfp* expression observed could be pseudotransduction of GFP protein or mRNA. This is unlikely because GFP fluorescence mediated by this phenomenon peaks at approximately 12 hours after infection and is hardly detectable after 48 hours [19]–[21]. Whether the detected titer reflects *gfp* expression from integrated or unintegrated lentiviral vector DNA is unknown. Thus, V^H^env and V^H^nef encoded transcripts could be packaged, reverse transcribed and transferred to target cells, although the vector titers were approximately 25 to 65-fold lower than those obtained for V^H^genomic.

### Encapsidation efficiencies

In order to analyze the influence of Rev on encapsidation of different lentiviral vector RNAs we extracted cytoplasmic and virion-associated RNA after cotransfection of HEK293T cells with the lentiviral vectors, expression plasmids for *tat*, VSV-G and *gag/gagpol* with or without a *rev* expression plasmid. The purity of the cytoplasmic fraction obtained with the mild lysis method used was rigorously tested previously [12], [13]. Virions from cell culture supernatants were purified through a 30% sucrose cushion. Cytoplasmic and particle RNA fractions were digested with DNase to remove remaining transfected plasmid DNA. Quantification of all RNA species was possible by specific quantitative RT-PCR protocols (see Materials and Methods S1, adapted from [9]). These PCRs do not detect the sequences of the *trans*-complementing *gag/gagpol, tat* and *rev* expression plasmids with relevant efficiency (see Materials and Methods S1). Control reactions without RT revealed efficient removal of the transfected plasmid DNA (data not shown).

**Figure 3:**
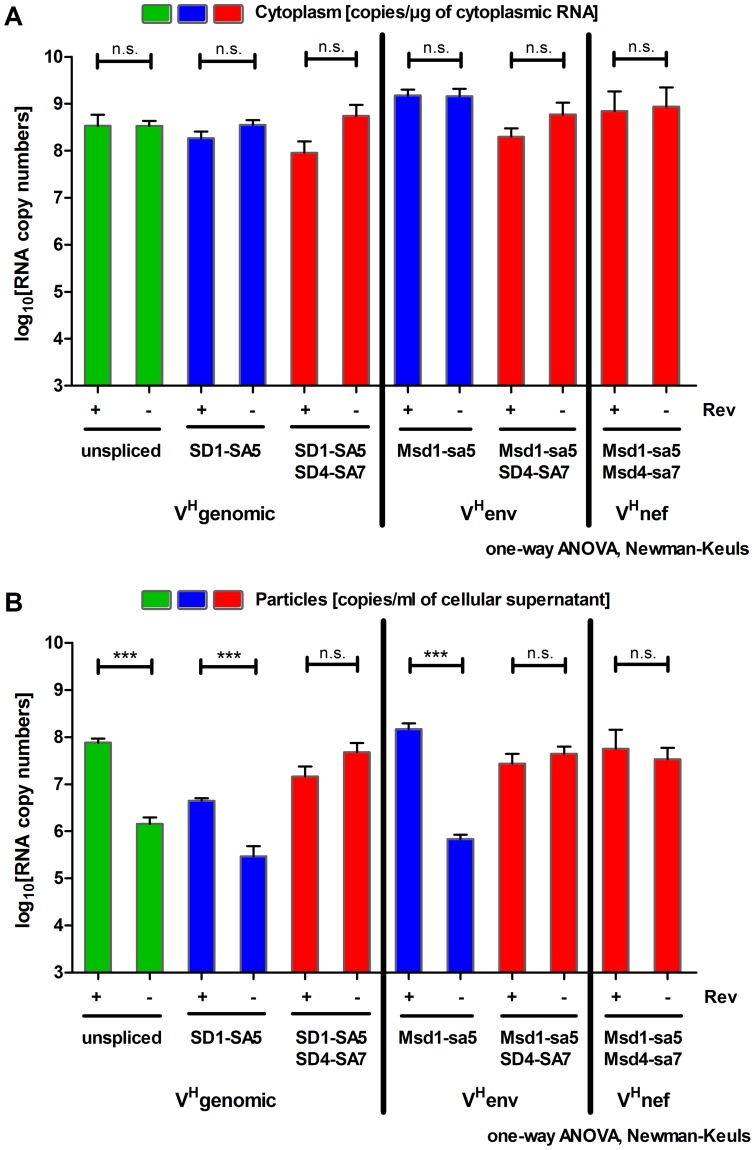
Cytoplasmic and virion-associated lentiviral vector RNA levels in the presence and absence of Rev. A) Cytoplasmic RNA was extracted two days after transfection and analyzed using quantitative RT-PCR protocols (please see Materials and Methods S1 for experimental details). Transcript copy numbers per µg of cytoplasmic RNA are shown. B) Virion-associated RNA was isolated from cell culture supernatants of cells analyzed in A. Transcript copy numbers per ml of cellular supernatant were obtained after RT-qPCR analyses. Unspliced RNA levels of V^H^genomic are shown in green. RNA levels of the singly-spliced SD1-SA5 RNA of V^H^genomic and the unspliced Msd1-sa5 transcript of V^H^env are depicted in blue. These RNAs represent the class of singly-spliced transcripts. Shown in red are transcript levels of the multiply-spliced SD1-SA5+SD4-SA7 RNA of V^H^genomic, the singly-spliced Msd1-sa5+SD4-SA7 RNA of V^H^env and the unspliced Msd1-sa5+Msd4-sa7 RNA of V^H^nef. These RNAs correspond to the class of fully-spliced transcripts. Mean values with SEM of log10 transformed RNA copy numbers obtained in 5 independent experiments are shown. Statistical analysis was performed with a one-way ANOVA combined with the Newman-Keuls post-test. ***, p≤0.001; **, p≤0.01; *, p≤0.05; n.s., not statistically significant.

**Figure 4:**
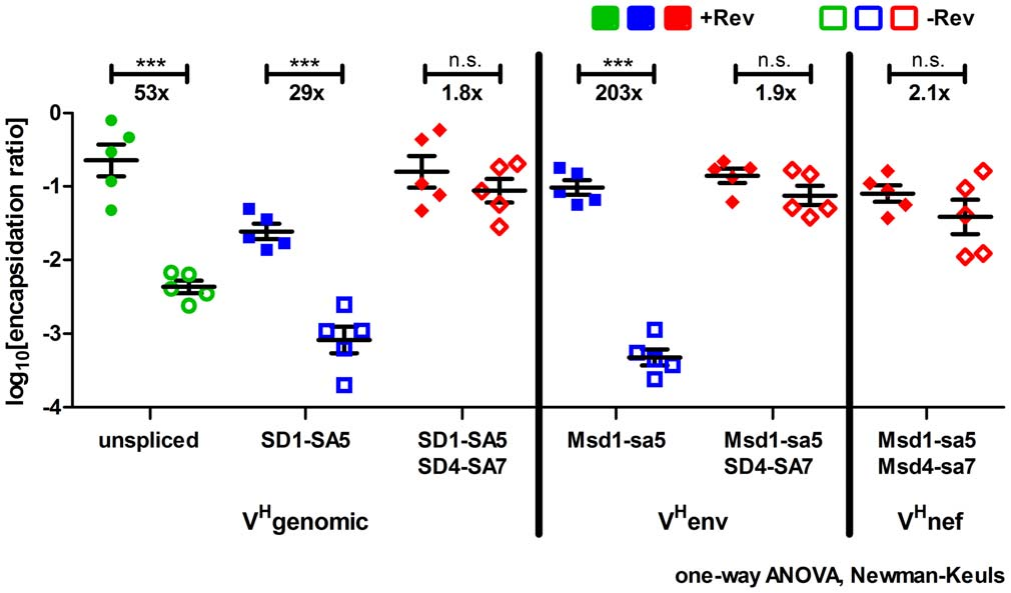
Encapsidation efficiency in the presence and absence of Rev. The ratio of virion-associated and cytoplasmic RNA levels defines the encapsidation efficiency for all lentiviral vector transcripts detected. The log10 transformed ratios were calculated for each single data pair obtained in each single experiment for all the different RNA species examined. Mean values with SEM obtained are shown. Statistical analysis was performed with a one-way ANOVA combined with the Newman-Keuls post-test. ***, p≤0.001; **, p≤0.01; *, p≤0.05; n.s., not statistically significant.

For all vector RNA species Rev had no statistically significant effect on cytoplasmic RNA levels (figure 3A). This is consistent with previous observations by us and others demonstrating that in the absence of Rev high amounts of unspliced lentiviral vector RNA are present in the cytoplasm of transfected cells and that adding Rev increased cytoplasmic vector RNA levels only minimally [13], [22]–[25]. This is unlikely due to large nuclear contamination of our cytoplasmic fraction, since the fractionation protocol was validated repeatedly in our previous publications [12], [13]. Nuclear proteins could not be detected in the cytoplasmic fraction by Western blotting and contamination of the cytoplasmic fraction with the nuclear pre-glyceraldehyde 3-phosphate dehydrogenase (pre-GAPDH) mRNA ranges from only 3 to 7% of the total amount of pre-GAPDH mRNA in the cell [12], [13]. Furthermore, the results obtained for the unspliced RNA of V^H^genomic in this work (figure 3 and 4) are fully consistent with the results we reported previously [13] confirming that the fractionation protocol worked as before. However, a fractionation control was not included in the particular experiments shown here. In contrast to observations with lentiviral vector constructs, Rev significantly enhances cytoplasmic RNA levels of wild type genomic HIV RNA. This difference between the genomic wild type and the lentiviral vector RNAs may be due to differences in their nuclear retention in the absence of Rev, since lentiviral vectors lack large regions of the HIV genome (see figure 1A) that are implicated in nuclear retention of viral RNA (*gag, pol* and *env* sequences). Previously, it could be shown that deletion or codon-optimization of these *cis*-acting sequences can reduce or prevent nuclear retention of the resulting transcripts even in the presence of splice donor and splice acceptor sites [26], [27]. In the present study no effect of Rev on cytoplasmic vector RNA levels could be observed. Assuming Rev-mediated nuclear RNA export at the expense of efficient Rev-independent export of these lentiviral vector RNAs could explain why Rev did not increase the cytoplasmic RNA levels of RRE-containing RNAs. In addition, the experimental variation for the determination of cytoplasmic copy numbers is too high to reveal more subtle changes. Strikingly different, a strong and differential effect of Rev on the amount of virion-associated RNAs could be observed (figure 3B). All RRE-containing transcripts were strongly enriched in virions when Rev was present. This effect varies between 30 and 200-fold and is statistically significant in all cases. In contrast, virion-associated RNA levels of all transcripts lacking an RRE did not vary significantly with or without Rev. In the presence of Rev the amount of particle-associated unspliced RNA of V^H^genomic was 17-fold and 5-fold higher compared to the levels of the singly-spliced SD1-SA5 RNA and the fully-spliced SD1-SA5+SD4-SA7 RNA, respectively. The unspliced RNA is therefore the predominant RNA species in viral particles. Remarkably, high amounts of unspliced RNAs of V^H^env and V^H^nef identical in sequence to the spliced transcripts of V^H^genomic could also be detected in viral particles. Consistent with this finding, packaging of an RNA mimicking the spliced HIV *env* transcript was previously shown by others but not quantified in detail [11].

The encapsidation efficiency was defined as ratio of virion-associated and cytoplasmic RNA levels. Mean values of log10 transformed ratios for each data pair of all repeat experiments for the RNA species analyzed are shown in figure 4. All RRE-containing transcripts showed a dramatic and statistically significant increase in their encapsidation efficiencies in the presence of Rev (figure 4). Since the encapsidation efficiency of a singly-spliced, RRE-containing HIV-1 *env* transcript expressed from a proviral HIV construct was similarly low as for the multiply-spliced *nef* transcript lacking the RRE, it was previously concluded that Rev does not influence packaging of HIV *env* RNA [9]. Our results clearly demonstrate that Rev is able to increase packaging of RRE-containing vector transcripts. This suggests that packaging of HIV *env* RNA could be inhibited by sequences not present in fully-spliced HIV RNAs. This negative effect could probably be overcome by a Rev-mediated nuclear export of *env* RNA leading to an encapsidation efficiency similar to that observed for the fully-spliced HIV transcript (see [9]).

A strong correlation between the Rev-dependent enhancement of the infectious vector titer (37-fold) and the encapsidation efficiency of the unspliced RNA of V^H^genomic (53-fold) confirms our previous results (figure 2 and 4 and [13]). Therefore, unspliced RNA of V^H^genomic exported to the cytoplasm in the absence of Rev cannot be efficiently encapsidated resulting in low viral titers. This could be explained by a direct or an indirect role of Rev in the encapsidation process. Binding of Rev to the RRE or to the Rev-binding site in the 5′UTR [28] could initiate the encapsidation process. However, such a direct effect is difficult to envision, because Rev was never reported to interact with HIV Gag and Rev is not known to be part of the HIV-virion. Therefore, the most plausible explanation is a more indirect effect such as the generation of an inhibitory ribonucleoprotein complex in the nucleus that prevents further cytoplasmic utilization of the intron-containing lentiviral vector RNA. A Rev-mediated nuclear export of this RNA would prevent/disrupt the association of inhibitory factors with the RNA and would allow cytoplasmic packaging to take place. Other possible explanations for a low efficient encapsidation process without Rev could be trapping of the RNA at a sub-cytoplasmic localization unfavorable for encapsidation or an inhibitory RNA structure. Nuclear export mediated by Rev could traffic the RNA to productive sub-cytoplasmic sites or prevent formation of inhibitory RNA structures thereby enabling efficient encapsidation by Gag [14].

Similar to the situation observed for V^H^genomic, Rev-dependent encapsidation of RRE-containing RNAs from V^H^env correlates with an enhanced infectious vector titer in the presence of Rev. However, the titer was increased by a factor of 6 whereas packaging of RRE-containing RNAs was enhanced by two orders of magnitude. Both the infectious titer and encapsidation of Msd1-sa5+Msd4-sa7 RNAs from V^H^nef lacking the RRE are not affected by Rev. It is evident that relative high amounts of unspliced and spliced vector RNAs are packaged after transfection of V^H^env and V^H^nef. Nevertheless, the infectious titer of both vectors in the presence of Rev is lower compared to V^H^genomic. These results imply that some steps after cell entry may be not as efficient for small vector transcripts compared to the unspliced transcript of V^H^genomic. These steps include the efficiency of reverse transcription, the formation of a functional preintegration complex, nuclear entry of the cDNA and finally integration into the cellular DNA.

The highest stimulatory effect of Rev could be observed on encapsidation of the RRE-containing Msd1-sa5 transcript encoded by V^H^env (figure 4, blue squares). Encapsidation of the transcript SD1-SA5 expressed from V^H^genomic identical in sequence to Msd1-sa5 was also increased by Rev but to a lesser extent (figure 4, blue squares). Competition between unspliced and spliced HIV transcripts was previously identified to diminish packaging of spliced RNAs [8], [9]. Therefore, a possible explanation for the efficient Rev-mediated packaging of Msd1-sa5 is the lack of such a competition between the different transcripts of V^H^env all of which do not contain the full-length encapsidation signal (figure 1 and figure 4). In contrast to the situation after transfection of V^H^env, V^H^genomic generates two transcripts, the unspliced and the singly-spliced SD1-SA5 RNA, which are exported to the cytoplasm by Rev. Since the unspliced transcript contains the full-length encapsidation signal that is truncated after splicing in the singly-spliced transcript, the presence of the unspliced RNA could limit encapsidation of the singly-spliced SD1-SA5 RNA.

Encapsidation efficiencies of lentiviral vector transcripts lacking the RRE did not vary significantly with or without Rev (figure 4, red diamonds). A 2-fold increase was observed in the presence of Rev for these RNAs. It is possible that this small stimulatory trend is mediated by binding of Rev to the first RNA stem loop in the encapsidation signal present in all viral transcripts [28]. Surprisingly, encapsidation of the fully-spliced lentiviral vector transcripts without RRE was highly efficient in our experiments (figure 4), while fully-spliced transcripts of wild type HIV are poorly packaged [9]. Furthermore, the amount of virion-associated genomic RNA in comparison to spliced RNAs is 20 to 40-fold higher in wild type HIV particles [10], [29], [30]. In our experiments this effect was smaller ranging from 5-fold for fully-spliced to 17-fold for singly-spliced RNAs (figure 3B). These facts demonstrate that the conditions in our experiments differed in some aspects from the wild type situation. A possible reason for these discrepancies could be that not all cells are cotransfected with every plasmid used. Cells transfected only with the Rev-independent *gag/gagpol* expression plasmid together with the lentiviral vector but not with the *rev* expression plasmid could lead to particles containing mainly fully-spliced RNAs, because packaging of Rev-dependent singly-spliced and unspliced RNAs would be impaired. In addition, high intracellular RNA levels obtained after transient transfection of HEK293T cells with the CMV promoter driven lentiviral vectors in the presence of Tat are believed to reduce the specificity of the encapsidation process [5]. Furthermore, utilization of the Rev and Tat-independent *gag/gagpol* expression plasmid could lead to increased packaging of fully-spliced RNA. In the HIV replication cycle the viral proteins Tat and Rev are expressed from multiply-spliced transcripts and have to accumulate to a certain threshold level to allow subsequent expression of genes from partially and unspliced RNAs [5], [31]–[34]. Additionally, the viral protein Gag/GagPol is produced from the unspliced transcript. These facts guarantee a spatial and temporal regulated appearance of Gag and genomic RNA at the same site in the cytoplasm late in the replication cycle. Most likely this concerted gene expression pattern allows an increased specificity of the encapsidation process. It is known that packaging *in trans* is efficient for HIV-1 and leads to infectious particles as observed in our experiments (figure 2 and 3). However, the specificity of this *trans*-packaging was to our knowledge never analyzed when a Rev- and Tat-independent *gag/gagpol* expression plasmid was used. Lentiviral vector production with such plasmids disconnects the spatial (*trans*-packaging) and temporal (Rev and Tat-dependency) regulation of the encapsidation process and could lead to an increased packaging of spliced RNA.

The splicing process itself could also result in a steric block of encapsidation, because the multi-protein exon-junction complex is deposited approximately 20 nt upstream of exon-exon junctions [35]. This complex could therefore occupy the residual 5′ part of the encapsidation signal in spliced RNAs and thus splicing itself could limit binding of Gag and packaging. Interestingly, packaging efficiencies of the singly-spliced SD1-SA5 RNA encoded by V^H^genomic and the unspliced Msd1-sa5 RNA expressed from V^H^env, which are identical in sequence, were similar (figure 4, blue squares). In the presence of Rev the mean encapsidation ratio of the unspliced Msd1-sa5 RNA is 4-fold higher than the ratio obtained for the spliced SD1-SA5 RNA (figure 4, blue filled squares, compare SD1-SA5 and Msd1-sa5 in the presence of Rev). However, in the absence of Rev the mean encapsidation ratio of the unspliced transcript is 2-fold lower (figure 4, blue open squares, compare SD1-SA5 and Msd1-sa5 in the absence of Rev). Furthermore, analyzing the mean values obtained for SD1-SA5 and Msd1-sa5 RNAs does not show statistically significant differences both in the presence and in the absence of Rev (one-way ANOVA with Newman-Keuls post-test, p>0.05). In addition, the unspliced Msd1-sa5+Msd4-sa7 and the corresponding singly-spliced Msd1-sa5+SD4-SA7 and fully-spliced SD1-SA5+SD4-SA7 RNAs show similar encapsidation efficiencies with and without Rev (figure 4, red diamonds). The mean encapsidation ratios of these spliced transcripts compared to the ratios of the unspliced transcript Msd1-sa5+Msd4-sa7 differed no more than 2-fold both in the presence and in the absence of Rev and these differences are not statistically significant (one-way ANOVA with Newman-Keuls post-test, p>0.05). Therefore, the splicing process itself does not seem to limit packaging of the vector RNAs analyzed.

## Conclusions

In conclusion, we could show that Rev clearly increases the encapsidation efficiency of unspliced and partially-spliced, RRE-containing HIV-vector transcripts. Furthermore, unspliced RNAs mimicking spliced vector transcripts can be packaged to a similar degree as their spliced counterparts with identical sequence arguing against a direct negative effect of splicing itself on encapsidation.

## Materials and Methods

### Plasmids

Expression plasmids for HIV-1 Rev (pcRev) [36], HIV-1 Tat (pcTat) [36], VSV-G (pHit/G) [37], HIV-1 Gag/GagPol (UTRgpRRE, Hgp^syn^) [18], [38] and the lentiviral vector HIV-CS-CG [16], [17] have been published elsewhere. V^H^genomic contains the first 339 nt of the HIV-1 HXB2 *gag* gene (nt 336 to 672 of GenBank entry NC_001802, frameshift mutation at ClaI site at nt 378). The sequence of HIV-1 *pol* is not present in this vector. An HIV-1 NL4.3 proviral fragment (nt 5915 to 6259 of GenBank entry AF324493) was inserted into the NotI site between SD1 and the RRE directly downstream of the remaining *gag* sequence. The first *rev* exon in this fragment was mutated to prevent expression (ATG to ATC: nt 5969 to 5971 and TAT to TAA: nt 6035 to 6037 of GenBank entry AF324493). All in all, 898 nt of *env* sequence including 39 nt of the 5′ *env* sequence as well as 859 nt containing the RRE, the SA7 and the splicing regulatory sequences ESE3, ESS3a and ESS3b are retained in V^H^genomic. The first intron between SD1 and SA5 spans 468 nt and the second intron between SD4 and SA7 comprises 981 nt. No viral proteins are expressed from this viral vector. To clone V^H^env and V^H^nef cytoplasmic RNA was extracted from HEK293T cells transfected with V^H^genomic and spliced transcripts were amplified by RT-PCR (QuantiTect Kit, Qiagen) as detailed in Materials and Methods S1. The resulting fragments as well as V^H^genomic were digested with KasI/NotI or KasI/EcoRI and ligated. In the resulting plasmid V^H^env and V^H^nef DNA sequences of SD1 and SA5 or SD1 and SA5 together with SD4 and SA7 are fused, respectively. All generated plasmids and PCR fragments were controlled by sequencing.

### Cell culture, transfections, Western Blot analyses and determination of infectious titers

HEK293 and HEK293T cells were cultured in Dulbecco's modified Eagle Medium (DMEM) with 10% fetal calf serum and appropriate antibiotics. Two days after cotransfection of HEK293T cells by the calcium phosphate coprecipitation method [18], [39] with the lentiviral vectors and *tat*, VSV-G and *gag/gagpol* expression plasmids with or without a *rev* expression plasmid the precleared (centrifugation for 5 min at 1,000 rpm) and filtered (0.45 µm filter) cell culture supernatant was used to infect HEK293 cells. Two days later green fluorescent cells were counted to obtain the infectious titer as GFP forming units per ml of supernatant (GFU/ml). Western Blot analyses were done essentially as described before [18] with the anti-CA antibody 183-H12-5C (NIH AIDS Research and Reference Reagent Program).

### Encapsidation assay and RT-qPCR

Determination of cytoplasmic and virion-associated RNA copy numbers of transfected HEK293T cells was done as described previously [12], [13], [18]. In brief, two days after transfection HEK293T cells were washed in cold PBS (Invitrogen) and cytoplasmic fractions were obtained by lysing the cells 5 min on ice in cold RLN buffer (50 mM Tris-Cl [pH 8.0], 140 mM NaCl, 1.5 mM MgCl_2_, 0.5% [vol/vol] Nonidet P-40, 1,000 U/ml RNase inhibitor, 1 mM dithiothreitol). Nuclei were pelleted by centrifugation for 2 min at 300xg and 4°C followed by careful collection of the supernatant as cytoplasmic fraction. Subsequently, the RNeasy Mini kit (Qiagen) was used to extract cytoplasmic RNA. Particle-associated RNA was prepared with the QIAamp Viral RNA Mini kit (Qiagen) after ultracentrifugation of the supernatants of transfected cells through a 30% sucrose cushion. All RNA samples were stored at −75°C until RT-qPCR analyses. Detection of unspliced RNA derived from V^H^genomic was facilitated by sense primer p2 binding upstream of SD1 (5′-gtggaaaatctctagcagtggcgc-3′) and antisense primer p4 binding directly downstream of SD1 (5′-tctttccccctggccttaaccg-3′). Detection of singly-spliced SD1-SA5 RNA of V^H^genomic and the unspliced Msd1-sa5 RNA of V^H^env was possible by sense primer p3- overlapping SD1 and SA5 (5′-ggggcggcgactggaagaa-3′) and antisense primer p8+ binding directly downstream of SD4 (5′-tgattactatggaccacacaactattgc-3′). The fully-spliced SD1-SA5+SD4-SA7 transcript of V^H^genomic and the corresponding transcripts with identical sequences Msd1-sa5+SD4-SA7 and Msd1-sa5+Msd4-sa7 of V^H^env and V^H^nef, respectively, were detected using sense primer p3- together with antisense primer p10 binding downstream of SA7 (5′-ccgttcactaatcgaatggatctgtc-3′). All primers are identical or very similar to those used by Houzet *et al*. [9]. RT-PCRs were done with 500 ng of cytoplasmic RNA and 1 µl from a 50 µl particle RNA fraction isolated from 5 ml of cell culture supernatant as described before [12], [13], [18]. For a detailed experimental description and the validation of the RT-qPCR approaches please see the Materials and Methods S1.

## Supporting Information

Materials and Methods S1 Detailed description of RT-(q)PCR approaches and validation of the methods including additional data.
(DOC)
