# Supplementary material for: The HIV-1 Rev Protein Enhances Encapsidation of Unspliced and Spliced, RRE-Containing Lentiviral Vector RNA
Source: PLoS One. 2012 Nov 1;7(11):e48688. doi: 10.1371/journal.pone.0048688 (PMC3486793; doi:10.1371/journal.pone.0048688)
Supplement: Materials and Methods S1 — Detailed description of RT-(q)PCR approaches and validation of the methods including additional data. (DOC) [file pone.0048688.s001.doc]

**Supplementary Materials and Methods S1**

**Detection and cloning of spliced transcripts**

Splicing of HIV-vector constructs was examined by Reverse Transcription (RT)-PCR. After transfection of V^H^genomic into HEK293T cells cytoplasmic RNA was extracted as described previously . The RT-PCR was done with 0.5 to 1 µg of isolated RNA together with the following primers which are depicted in figure 1B of the main manuscript (arrowheads). The sense primer overlaps with the 3’ end of the R region and the primer binding site upstream of SD1 (nt 616 to 638 of GenBank entry K03455 - sense 5’-gtgtggaaaatctctagcagtgg-3’). Antisense primer 1 binds downstream of SD4 but upstream of SA7 (nt 7622 to 7637 of GenBank entry K03455 and three vector specific nt underlined - antisense1 5’-tccaggtctgaagatcagc-3’). The second antisense primer binds downstream of SA7 in the 5’ end of the CMV-GFP expression cassette (antisense2 5’-tgaactaatgaccccgtaattga-3’). RT reactions were done at 50°C for 30 min with the QuantiTect Probe RT-PCR Kit (Qiagen) followed by 15 min of 95°C. Subsequently, 30 PCR cycles started at 95°C for 30 s, 55°C for 30 s and 72°C for 30 s/500 bp of amplicon length. A final extension step was done at 72°C for 7 min. With the combination of the sense primer with antisense primer 1 possible amplicons are 905 bp or 437 bp in length corresponding to the unspliced RNA or the singly-spliced SD1-SA5 RNA of V^H^genomic, respectively. The fully-spliced SD1-SA5+SD4-SA7 RNA cannot be detected because the binding site of antisense primer 1 is deleted by splicing of the second intron. The sense primer in combination with the second antisense primer can result in fragments of 1801 bp, 1333 bp or 352 bp in length corresponding to the unspliced RNA, the singly-spliced SD1-SA5 RNA or the fully-spliced SD1-SA5+SD4-SA7 RNA, respectively. Short elongation steps were chosen to preferentially detect spliced transcripts.

To clone the vectors mimicking the spliced transcripts, the RT-PCR products were separated by agarose gel electrophoresis. Amplicons corresponding to the singly-spliced SD1-SA5 RNA and the fully-spliced SD1-SA5+SD4-SA7 RNA were extracted and digested with the restriction enzymes KasI/NotI or KasI/EcoRI, respectively. Ligation with the plasmid DNA of V^H^genomic digested with the same enzymes generated plasmids V^H^env and V^H^nef in which sequences of SD1 and SA5 or SD1 and SA5 together with SD4 and SA7 are fused, respectively. Cytoplasmic RNA was also extracted after transfection of V^H^env and V^H^nef and RT-PCRs were done as described above. All generated plasmids and PCR fragments were controlled by sequencing.

**Quantitative Reverse Transcription-PCR**

Cytoplasmic and virion-associated RNA were isolated as described previously . Detection of unspliced RNA derived from V^H^genomic was facilitated by sense primer p2 overlapping with the 3’ end of the R region and the primer binding site upstream of splice donor 1 (SD1) (nt 618 to 641 of GenBank entry K03455 - 5’-gtggaaaatctctagcagtggcgc-3’) and antisense primer p4 binding directly downstream of SD1 (nt 847 to 868 of GenBank entry K03455 - 5’-tctttccccctggccttaaccg-3’). Detection of singly-spliced SD1-SA5 RNA of V^H^genomic and the unspliced Msd1-sa5 RNA of V^H^env was possible by sense primer p3- overlapping SD1 and splice acceptor 5 (SA5) (nt 731 to 743 and nt 5977 to 5982 of GenBank entry K03455 - 5’-ggggcggcgactggaagaa-3’) and antisense primer p8+ binding directly downstream of SD4 (nt 6116 to 6143 of GenBank entry K03455 - 5’-tgattactatggaccacacaactattgc-3’). The fully-spliced SD1-SA5+SD4-SA7 transcript of V^H^genomic and the corresponding transcripts with identical sequences Msd1-sa5+SD4-SA7 and Msd1-sa5+Msd4-sa7 of V^H^env and V^H^nef, respectively, were detected using sense primer p3- (see above) together with antisense primer p10 binding downstream of SA7 (nt 8451 to 8476 of GenBank entry K03455 - 5’-ccgttcactaatcgaatggatctgtc-3’). All primers are identical or very similar to those used by Houzet *et al*. . Amplicons obtained with combinations of primers p2/p4, p3-/p8+ or p3-/p10 are 253 bp, 177 bp or 180 bp in length, respectively. Amplification of the 1161 bp fragment with primers p3- and p10 (e.g. singly-spliced SD1-SA5 RNA of V^H^genomic) was prevented by short elongation times.

Standard curves were generated in all RT-qPCR experiments using *in vitro* transcribed RNA. These RNAs were prepared with the AmpliScribe-T7/SP6 High Yield Transcription Kit (Epicentre) using amplicons containing the three different transcripts and the Sp6 or T7 promoter. The RNA was precipitated with ammonium acetate and treated with the MBU-DNaseI (AmpliScribe-T7/SP6 High Yield Transcription Kit, Epicentre). Complete removal of all input DNA was achieved by two additional DNase treatments with the DNA-free DNase treatment and removal kit (Ambion). The RNA concentration was determined with the RiboGreen RNA Quantification Kit (Invitrogen) in a RotorGene 3000TM (Corbett Research). The RNA was diluted in water to obtain 10^9^ specific RNA copies per µl. Serial 10-fold dilutions in 0.5 µg/µl cytoplasmic RNA solution from untransfected cells resulted in RNA standards from 10^8^ down to 10^3^ specific RNA copies per µl.

RT-qPCRs were prepared with the QuantiTect Probe RT-PCR Kit (Qiagen) in combination with the intercalating dye SYBRGreen (Invitrogen). The RT-qPCRs were performed with 500 ng of cytoplasmic RNA or 1 µl from a 50 µl particle RNA fraction isolated from 5 ml of cell culture supernatant as described before . All RT-qPCRs were performed in a RotorGene TM3000 (Corbett Research) under the conditions summarized in Supplemental Table S1. The unspliced RNA of V^H^genomic was detected with primer pair p2/p4 and fluorescence measurement at 84°C. The SD1-SA5 RNA of V^H^genomic and the Msd1-sa5 RNA of V^H^env were detected with primer pair p3-/p8+ and fluorescence readout at 80°C. The SD1-SA5+SD4-SA7 RNA of V^H^genomic, the Msd1-sa5+SD4-SA7 RNA of V^H^env and the Msd1-sa5+Msd4-sa7 RNA of V^H^nef were analyzed with primers p3-/p10 and fluorescence of the specific amplicon was recorded at 81°C. The *in vitro* transcribed RNAs with concentration from 10^3^ to 10^9^ RNA copies per reaction were measured in parallel and resulted in linear standard curves from 10^3^/10^4^ to 10^9^ RNA copies (Supplemental Figure S1, S2

Supplemental Table S1: RT-qPCR protocols


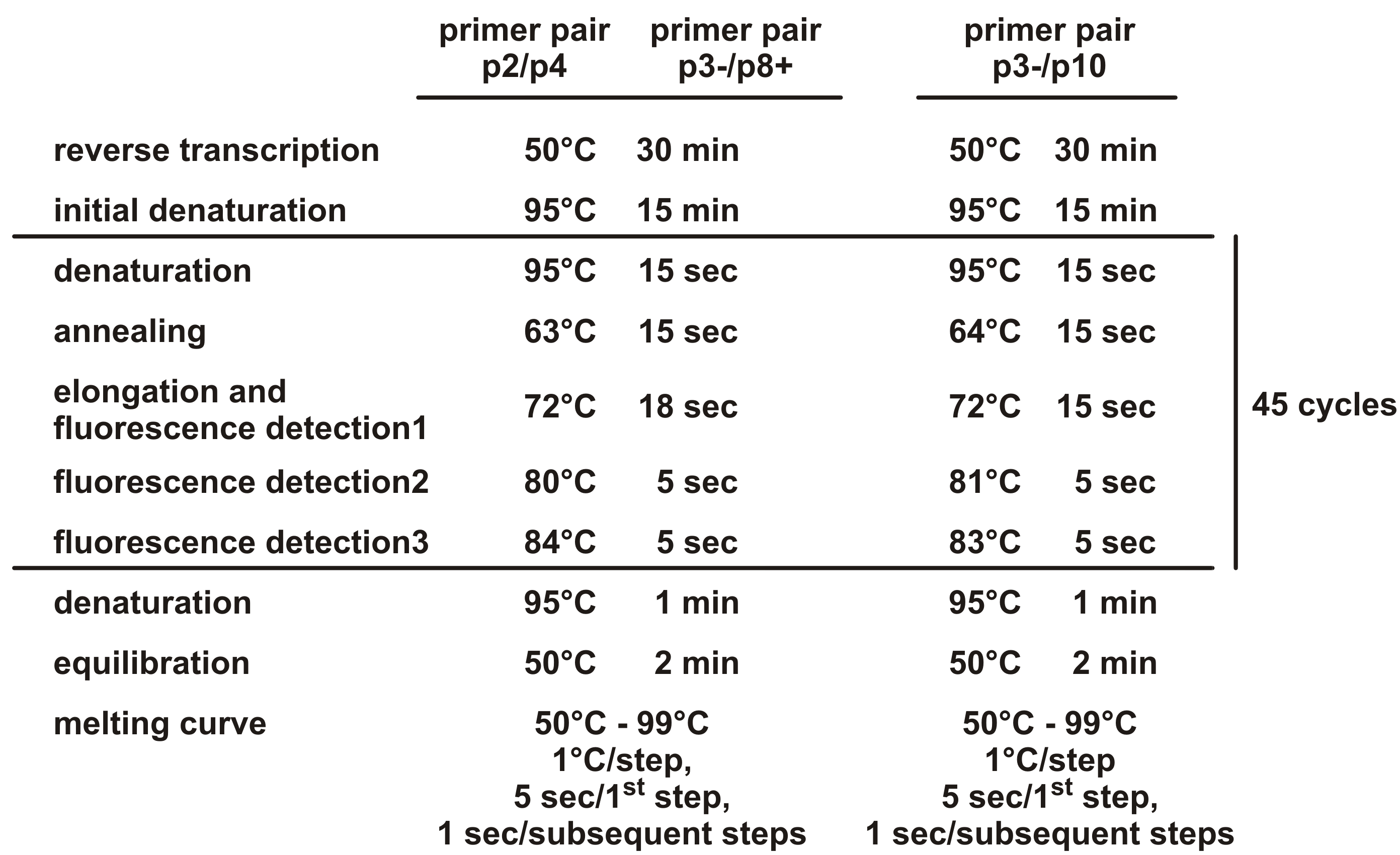


and S3). Copy numbers per µg of cytoplasmic RNA or per ml of analyzed cell culture supernatants were calculated and mean values of log10 transformed copy numbers are reported. Detection of primer-dimers was excluded by fluorescence measurements at elevated temperatures and by melting curve analyses in each run. In addition, the specificity of the RT-qPCRs was confirmed by agarose gel electrophoresis of the amplicons regularly throughout the duration of the project. Control reactions without RT were also performed regularly and demonstrated the successful removal of transfected plasmid DNA by the DNase treatment with the DNA-free DNase treatment and removal kit (Ambion).

**Validation of the specificity of the RT-qPCRs**

Only amplicons of the expected lengths were detected by agarose gel electrophoresis after RT-qPCRs of cytoplasmic RNA from cells transfected with V^H^genomic, V^H^env or V^H^nef for the analyses of encapsidation efficiencies. RT-qPCRs with cytoplasmic RNA from untransfected cells remained negative (data not shown). Since different lentiviral vector transcripts are present in the cells after transfection, it was important to verify that only the specific RNA was detected by the matched primer pair. The specificity of the amplification process was therefore controlled by a PCR experiment with the template plasmid DNA of V^H^genomic, V^H^env and V^H^nef mimicking the unspliced RNA, the singly-spliced RNA and the fully-spliced RNA, respectively (Supplemental Figure S4). All three primer pairs detected only the corresponding target sequence. No amplification products could be seen with unmatched primer-DNA pairs. Additional qPCR assays revealed that the detection efficiency for V^H^genomic, V^H^env or V^H^nef with matched primer pairs was at least 10^9^-fold higher than for V^H^genomic, V^H^env or V^H^nef with unmatched primer pairs. Adding 10^9^ copies of Hgp^syn^ to the qPCR assay did not reveal any background signal for primer pair p3-/p8+ and p3-/p10. For primer pair p2/p4 a weak signal equivalent to 1300 copies of V^H^genomic was observed. This indicates that the detection efficiency of the qPCR for the transcripts of Hgp^syn^ is approximately 10^6^-fold lower than for V^H^genomic. Since cellular transcript levels of Hgp^syn^ and V^H^genomic should be of the same order of magnitude, Hgp^syn^ cannot affect the determination of the vector RNA copy numbers. Similar experiments were performed with pcRev and pcTat revealing that the detection efficiency of the three qPCR assays was at least 10^8^-fold lower than for their specific lentiviral vector target.

**A**

**
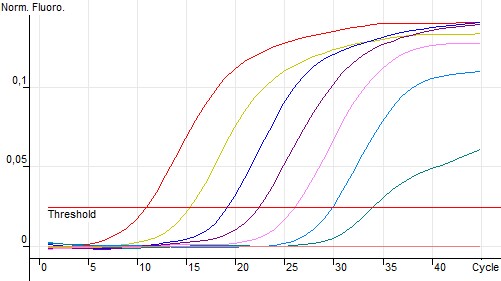
**

**B**

**
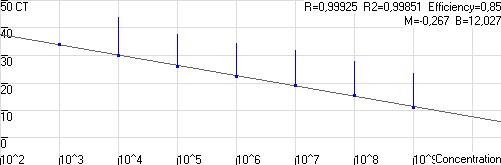
**

Supplemental Figure S1: Linearity of standard curve after amplification with primer pair p2/p4

**A)** The SYBRGreen fluorescence detected at 84°C in an RT-qPCR with experimental settings depicted in table S1 and *in vitro* transcribed RNA standards as template is shown. The standards encompass the sequence amplified by the primer pair p2/p4. **B)** A linear correlation between the Ct values and the RNA copy numbers in a range of 10^9^ and 10^3^ standard RNA copies per reaction could be observed. The melting curve analysis demonstrated a melting temperature of 91.4 to 91.6°C of the specific amplicon whereas the negative water sample showed an amplicon (most probable primer dimers) with a melting temperature of 81.3°C (data not shown). The amplicon in the water sample was not efficiently detected at a temperature of 84°C that was used to detect the SYBRGreen fluorescence.

**A**

**
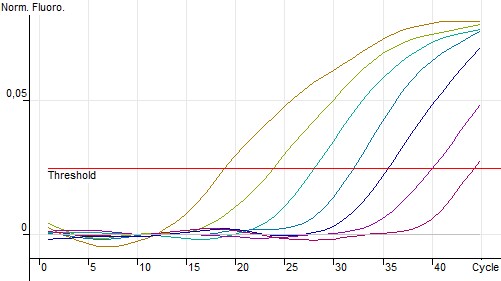
**

**B**

**
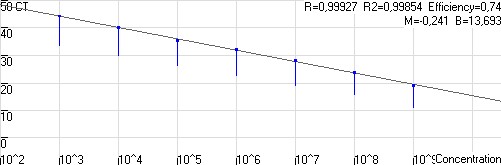
**

Supplemental Figure S2: Linearity of standard curve after amplification with primer pair p3-/p8+

**A)** The SYBRGreen fluorescence detected at 80°C in an RT-qPCR with experimental settings depicted in table S1 and *in vitro* transcribed RNA standards as template is shown. The standards encompass the sequence amplified by the primer pair p3-/p8+. **B)** A linear correlation between the Ct values and the RNA copy numbers in a range of 10^9^ and 10^3^ standard RNA copies per reaction could be observed. The melting curve analysis demonstrated a melting temperature of 84.6 to 85.1°C of the specific amplicon whereas the negative water sample showed an amplicon (most probable primer dimers) with a melting temperature of 81.1°C (data not shown). The amplicon in the water sample was not efficiently detected at a temperature of 80°C that was used to detect the SYBRGreen fluorescence.

**A**

**
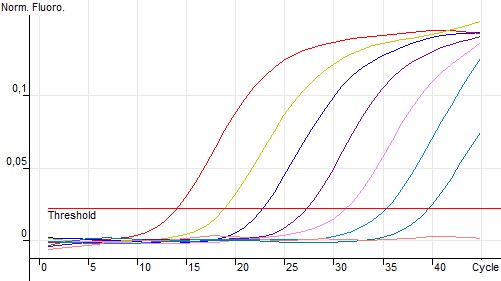
**

**B**

**
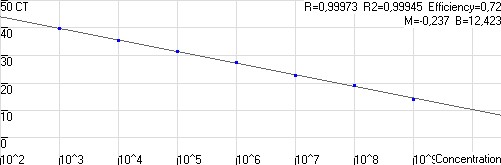
**

Supplemental Figure S3: Linearity of standard curve after amplification with primer pair p3-/p10

**A)** The SYBRGreen fluorescence detected at 81°C in an RT-qPCR with experimental settings depicted in table S1 and *in vitro* transcribed RNA standards as template is shown. The standards encompass the sequence amplified by the primer pair p3-/p10. **B)** A linear correlation between the Ct values and the RNA copy numbers in a range of 10^9^ and 10^3^ standard RNA copies per reaction could be observed. The melting curve analysis demonstrated a melting temperature of 88.1 to 88.6°C of the specific amplicon whereas the negative water sample showed an amplicon (most probable primer dimers) with a melting temperature of 79.9°C (data not shown). The amplicon in the water sample was not efficiently detected at a temperature of 81°C that was used to detect the SYBRGreen fluorescence.


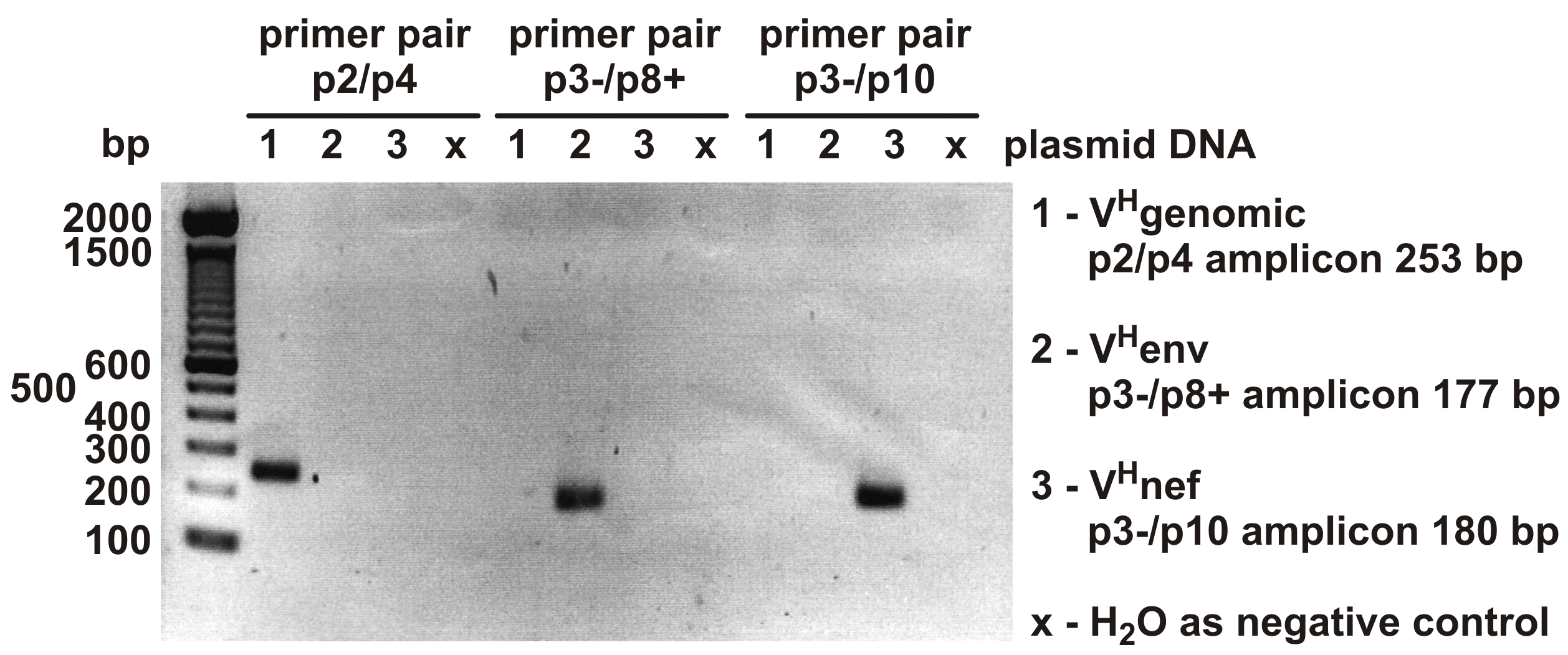


Supplemental Figure S4: PCR specificity

DNA fragments obtained after amplification of 10 ng of the plasmids V^H^genomic, V^H^env or V^H^nef in PCRs with the primer pairs p2/p4, p3-/p8+ and p3-/p10 under experimental settings similar to those shown in table S1 (differences: no RT step, no fluorescence detections, no melting curve analyses, 40 cycles) were separated by agarose gel electrophoresis. Only matched DNA-primer pairs resulted in specific amplicons.

**References cited in the supplementary materials and methods S1**

1. Blissenbach M, Grewe B, Hoffmann B, Brandt S, Uberla K (2010) Nuclear RNA export and packaging functions of HIV-1 Rev revisited. J Virol 84: 6598-6604.

2. Brandt S, Blissenbach M, Grewe B, Konietzny R, Grunwald T, et al. (2007) Rev proteins of human and simian immunodeficiency virus enhance RNA encapsidation. PLoS Pathog 3: e54.

3. Grewe B, Hoffmann B, Ohs I, Blissenbach M, Brandt S, et al. (2012) Cytoplasmic utilization of human immunodeficiency virus type 1 genomic RNA is not dependent on a nuclear interaction with Gag. J Virol 86: 2990-3002.

4. Lassen KG, Ramyar KX, Bailey JR, Zhou Y, Siliciano RF (2006) Nuclear retention of multiply spliced HIV-1 RNA in resting CD4+ T cells. PLoS Pathog 2: e68.

5. Grewe B, Überla K (2010) Rev Revisited: Additional Functions of the HIV-1 Rev Protein. In: Lever AML, Jeang K-T, Berkhout B, editors. Recent Adcances in Human Retroviruses: Principles of Replication and Pathogenesis. Singapore: World Scientific Publishing Co. Pte. Ltd. pp. 439-470.

6. Houzet L, Paillart JC, Smagulova F, Maurel S, Morichaud Z, et al. (2007) HIV controls the selective packaging of genomic, spliced viral and cellular RNAs into virions through different mechanisms. Nucleic Acids Res 35: 2695-2704.
